# Project-of-being in transformation: existential experience in bariatric surgery in light of phenomenology

**DOI:** 10.1590/0034-7167-2025-0344

**Published:** 2026-07-31

**Authors:** Caroline Linhares de Jesus, Luciara Fabiane Sebold, Bárbara Mohr da Silveira, Lucia Nazareth Amante, Helena Moraes Cortes, Nadia Maria Chiodelli Salum

**Affiliations:** I Universidade Federal de Santa Catarina. Florianópolis, Santa Catarina, Brazil; II Hospital Universtiário Professor Polydoro Ernani de São Thiago. Florianópolis, Santa Catarina, Brazil

**Keywords:** Obesity, Bariatric Surgery, Nursing, Mental Health, Philosophy., Obesidad, Cirugía Bariátrica, Enfermería, Salud Mental, Filosofía.

## Abstract

**Objectives:**

to understand, in light of the Heideggerian hermeneutic phenomenology, the existential experiences of people who have undergone bariatric surgery.

**Methods:**

qualitative study conducted between April and July 2024 with 13 participants at different stages of postoperative recovery, recruited via social media and snowball sampling. Interviews were conducted remotely and analyzed in light of Heidegger’s hermeneutic phenomenology.

**Results:**

the narratives indicate that obesity is experienced as an existential limitation that affects identity, social relationships, and being-in-the-world. Surgery emerges as a possibility for reframing existence, but it can cause compulsions, emotional challenges, and estrangement from the transformed body.

**Conclusions:**

bariatric surgery is experienced as an ontological phenomenon of self-transformation. Multidisciplinary care, with an existential and psychological focus, is essential to support the reconstruction of the project of being and the relationship with the lived body.

## RESUMEN

**Objetivos:**

comprender, a la luz de la fenomenología hermenéutica heideggeriana, las experiencias existenciales de las personas sometidas a cirugías bariátricas.

**Métodos:**

estudio cualitativo realizado entre abril y julio de 2024, con 13 participantes en diferentes etapas del postoperatorio, reclutados a través de redes sociales y mediante muestreo de “bola de nieve”. Las entrevistas se realizaron de forma remota y se analizaron a la luz de la fenomenología hermenéutica de Heidegger.

**Resultados:**

las narrativas indican que la obesidad se vive como una limitación existencial que afecta a la identidad, a las relaciones sociales y al ser en el mundo. La cirugía surge como una posibilidad de reinterpretar la existencia, pero puede provocar compulsiones, retos emocionales y extrañamiento ante el cuerpo transformado.

**Conclusiones:**

la cirugía bariátrica se vive como un fenómeno ontológico de transformación del ser. La atención multiprofesional, con un enfoque existencial y psicológico, es esencial para apoyar la reconstrucción del proyecto del ser y de la relación con el cuerpo vivido.

## INTRODUCTION

In recent decades, obesity has become a public health issue of pandemic proportions, affecting millions of people worldwide^(1)^. This increase reflects a complex interaction among changes in the food environment, sedentary lifestyles, socioeconomic, environmental, and genetic factors, a phenomenon that goes beyond simplistic explanations^(2)^. Obesogenic environments have a direct impact on behavioral choices and contribute significantly to the spread of this condition^(1)^.

Therefore, given its complexity, obesity requires an integrated and intersectoral approach involving health professionals, industry, social organizations, and, above all, public authorities, whose leadership in the formulation and implementation of evidence-based policies is essential to effectively address the problem^(3,4)^. This coordination is essential to address structural challenges and promote sustainable interventions that take into account the social determinants of health.

According to the Brazilian Association for the Study of Obesity and Metabolic Syndrome (ABESO) and the Brazilian Society of Endocrinology and Metabolism (SBEM), obesity is characterized by excessive accumulation of body fat, with the body mass index (BMI) being the main diagnostic parameter. Overweight corresponds to a BMI between 25.0 and 29.9 kg/m^2^, obesity is defined as a BMI ≥30 kg/m^2^, and severe obesity as a BMI ≥40 kg/m^2^ or ≥35 kg/m^2^ with comorbidities. However, new guidelines propose supplementing this classification by considering the maximum weight achieved in life (MWAL). This approach values modest weight loss, capable of providing clinical benefits even without leaving the obesity range, favoring a more individualized management^(5,6)^.

Although widely adopted, BMI alone does not take into account the complexity of the diagnosis. Therefore, for a more accurate assessment, it is necessary to consider additional factors such as body composition, fat distribution, ethnicity, presence of comorbidities, and clinical history, in addition to the use of complementary methods such as bioimpedance^(6-8)^.

The recognition of obesity as a risk factor for chronic diseases such as type 2 diabetes, hypertension, and cardiovascular disease requires not only psychosocial changes but also effective therapeutic strategies^(9,10)^. Treatment begins with lifestyle changes, including diet, physical activity, psychological support, and, if necessary, medication. However, in cases of severe obesity that do not respond to conservative approaches, bariatric surgery is recommended^(7,11)^.

Such surgery, however, is not limited to a technical procedure to modify the gastrointestinal tract. It represents an ontological rupture in the way the subject inhabits their own body and relates to the world^(12)^. Physical and metabolic changes trigger transformation processes that affect not only the biological body, but also the identity, affective, relational, and existential spheres. From Heidegger’s phenomenological perspective, obesity is understood as an experience that conditions and shapes being-in-the-world, with direct interference in the subject’s existential project, in the possibilities of being, and in the way they launch themselves into the world.

The obese body is not just a biological body. It is lived and experienced as a body laden with meanings, often associated with stigma, inadequacy, and isolation. The limitations imposed by reduced mobility, associated with social and symbolic barriers, become experiences that profoundly affect the constitution of these people’s project-of-being. Heidegger warns us that being is not given, but is always in process, in constant unfolding, traversed by temporality, historicity, and interpersonal relationships^(13)^.

Causal linearity and scientific neutrality, in this context, prove insufficient to explain the experience of obesity and the post-surgical transformation process. The complexity does not lie in the object itself, but emerges from the relationship between the subject and the world, in which knowing is, above all, a being-with, an involvement. Bariatric surgery, then, not only alters the physical configuration of the body, but also calls on the subject to redefine their own existence, crossing boundaries between the lived body and the objectified body.

On the one hand, if weight loss favors social reintegration, increased mobility, and improved health, on the other hand, it can cause feelings of strangeness in the face of a body that presents itself as another, different from the one that has been lived for so long. This process often brings emotional tensions to the surface, such as anxiety, depression, body dissatisfaction, and, not rarely, the emergence or worsening of eating disorders, as a result of a fragile relationship with one’s own image and with food^(14,15)^.

Heideggerian phenomenology shifts the focus from epistemology to ontology, allowing us to understand that the experience of bariatric surgery is not simply that of a successful or failed medical procedure, but rather that of becoming, constantly under construction. Heidegger invites us to think that being is not an object at the disposal of consciousness, but an ever-unfinished possibility, traversed by time, language, and openness to the world. In this sense, understanding the experience of obesity and surgery is, above all, opening oneself to lived experience, to the ways in which subjects perceive themselves and build their worlds.

Given this scenario, the present study, grounded in Heidegger’s hermeneutic phenomenology, seeks to understand the emotional and existential aspects people experience during bariatric surgery, from the decision to undergo the procedure to adaptation to the new condition of being-in-the-world. To this end, the following questions were developed: How do patients perceive their condition of obesity in the context of being-in-the-world? What meanings emerge during the decision-making process and adaptation to this surgery? How do bodily transformations impact these people’s project-of-being?

## OBJECTIVES

To understand, in light of Heideggerian hermeneutic phenomenology, the existential experiences of people who have undergone bariatric surgery.

## METHODS

### Ethical aspects

The study was conducted in accordance with national and international ethical guidelines and approved by the Ethics Committee of the Federal University of Santa Catarina, whose opinion is attached to this submission. All participants signed a Free and Informed Consent Form (FICF) and, to ensure anonymity and confidentiality, were identified by the letter “P,” followed by a sequential number corresponding to the order in which the interviews were conducted (P1, P2, ..., P13).

### Study design

This is a qualitative study based on Heideggerian hermeneutic phenomenology (2015). The aim was to understand the meanings and significance people attribute to the experience of bariatric surgery, considering the physical, emotional, and existential transformations that shape their ways of being in the world. Hermeneutic phenomenology^(13)^ provides access to lived experience through an understanding of being as possibility, in constant unfolding and construction.

### Methodological procedures

Both the organization and description of the study followed the guidelines of the Consolidated Criteria for Reporting Qualitative Research (COREQ)^(16)^ to ensure transparency, completeness, and quality of reports in qualitative research, particularly with regard to methodological design, participant recruitment, and data collection and analysis.

The inclusion criteria were: adults (over 18 years of age) residing in the state capital who underwent bariatric surgery at different stages, more specifically during the immediate postoperative period (up to 3 months), intermediate postoperative period (3 to 12 months), and late postoperative period (over 12 months), and who were cognitively and emotionally able to participate in the interview. People in the preoperative period were excluded, as well as those with cognitive limitations that compromised verbal communication and understanding of the questions.

Recruitment began on Instagram via the researcher’s private network, with posts and stories inviting people to participate in the research and a link to contact via WhatsApp to ensure privacy. From then on, people who contacted us and accepted the invitation were asked to refer others who had also undergone bariatric surgery, which characterizes the “snowball sampling” technique and favors diversity of experiences. The number of participants was determined according to the principle of theoretical saturation, i.e., the inclusion of new participants was discontinued when the data began to show redundancy and no new elements relevant to a deeper understanding of the phenomenon emerged^(17)^. The final sample consisted of 13 participants, a number considered sufficient for phenomenological analysis.

### Study setting

The study was conducted between April and July 2024 in a capital city in southern Brazil.

### Data collection and organization

The data were collected through individual, semi-structured interviews conducted remotely via Google Meet, according to the participants’ availability. The Informed Consent Form (ICF) was sent and explained in advance via WhatsApp, ensuring ethical understanding. A semi-structured script with 15 open-ended questions was used to identify, based on the participants’ reports, their personal experiences related to bariatric surgery, including positive and negative feelings, difficulties faced, facilitating factors, and available support network. The interviews, which lasted an average of 40 minutes, were recorded and transcribed in full for analysis. The researcher in charge had prior experience with qualitative interviews, ensuring ethical conduct and adherence to phenomenological rigor. The interviews were not validated by the participants.

### Data analysis

The data analysis followed the principles of Heideggerian hermeneutic phenomenology, which seeks to understand participants’ ways of being-in-the-world through their narratives. This interpretive approach is not limited to the description of phenomena but aims to reveal the meanings that emerge in discourse, understanding being as an open possibility in constant relation to the world^(13)^.

The analysis took place in three stages: exhaustive reading of the transcripts to gain an overall understanding of the experiences; identification of units of meaning related to existential categories such as being-in-the-world, lived body, and care; and hermeneutic analysis to articulate text and existential context and thus reveal deeper meanings in accordance with Heideggerian ontology^(18)^. The phenomenological hermeneutic view presupposes circularity in analysis, such that understanding a part of the discourse refers to the totality of the experience, which in turn reinterprets each part, in a movement known as the hermeneutic circle^(13,19)^.

## RESULTS

The study involved 13 people at different stages of post-operative recovery after bariatric surgery, seven women and six men, aged 30 to 60. The time elapsed since the surgical procedure varied widely: from participants who had been operated on 10 days ago to those who had been recovering for 8 years.

Based on the analysis of the data obtained, four categories emerged: Understanding the body as a limitation and a possibility; Between anguish and hope: the being facing the first experiences in the post-bariatric period; Being in transition: between the strangeness of the body and the psycho-emotional challenges in the post-bariatric period; and Reframing the body, rebuilding the being: the transformation of self-esteem after bariatric surgery.

The categories summarizing the challenges faced in the perioperative stages of bariatric surgery are illustrated below through participants’ testimonials. They reveal their experiences, with their meanings, in relation to the bariatric surgery process.

### Understanding the body as a limitation and a possibility

Dissatisfaction with body image was experienced by participants as a rupture in their relationships with the world, in which the obese body became a ‘body-object’, an obstacle to their project of being. Frustrated attempts to lose weight reveal a quest to regain control over one’s own body and reaffirm identity, but in a process generally permeated by failures and an intensification of feelings of alienation from the world and from oneself.

*Frustration at not being able to lose weight, low self-esteem, especially when I saw myself in photos.* (P1)

*Before the surgery, I always felt sad, with no motivation to do anything, lots of frustration at trying to lose weight and not succeeding, frustration on special occasions or at parties like weddings, where I had to buy clothes and nothing fit me.* (P3)

*Frustration defines it. I didn’t wear the clothes I liked, but rather the ones that fit me. Not to mention the bullying I suffered. We don’t have your size.* (P7)

Another aspect that was frequently mentioned was concern for health. Some attributed the decision to undergo surgery to the worsening of their clinical condition, which made them aware of the urgency of surgical intervention.

*Initially, I didn’t want to have the surgery, but my physical health was deteriorating and I needed to lose weight. I set a goal of losing one kilogram per month over one year, under the supervision of an endocrinologist, nutritionist, and psychologist. Anyway, after a year, I didn’t reach my goal, so I opted for surgery.* (P5)

*Every time I went to a doctor or nutritionist and saw my weight increasing and my test results worsening, I saw myself with less of a future. One of the most relevant factors that made me decide to have the surgery was seeing that, at 26, I had the same diseases my father had at 70. That was crucial to my decision.* (P9)

*I felt a constant lack of energy, memory problems, and relationship problems with my family because of my obesity and sleep apnea. With the changes that preceded my surgery* [abstinence from alcohol, exercise routine]*, I lost 8 kg in one month. This made me even more motivated to have the surgery.* (P12)

Depression and physical/mental fatigue were also identified as emotional factors present before surgery:

*Feeling obese seemed to be taboo, not very well accepted in society or in relationships. I also felt more depressed and tired, mentally and physically.* (P10)

It is possible to understand, from the narratives, that obesity is not merely a biomedical condition but presents itself as an existential experience marked by suffering, frustration, anguish, and, simultaneously, a desire for transformation. The body, experienced as a limitation due to aesthetic dissatisfaction and functional restrictions or health impacts, also emerged as a possibility, mobilizing the subjects to redefine their own existence.

### Between anguish and hope: the being facing the first experiences in the post-bariatric period

In the period immediately following surgery, participants experienced a range of conflicting emotions, which can be interpreted as reflections of the attempt to open up to being-in-the-world. Anxiety and fear represent existential anguish in the face of new possibilities and responsibilities that emerged with the transformed body, while hope and expectations reflect a movement toward a new project of being.

*Yes, after the surgery, I regretted it during the liquid phase, but after that phase, it passed, and I became more nervous about not being able to eat solids. After a month, my mind was completely balanced as usual.* (P2)

[...] *I went through complications. So, for the first two months, I just felt regret. I thought I was going to die.* (P3)

*I felt a little fragile, I felt humiliated, like I needed help from other people, and I thought, my God, why didn’t I take care of my weight before? Of myself, in general, so I wouldn’t have to go through this.* (P9)

*The first few weeks, let’s say about 30 days later, when I started eating solids, I felt like I would never be able to eat normally again. During this period, you vomit and your stomach hurts, and then you regret that you will never be able to eat solids again without suffering, but it is temporary.* (P10)

Some participants experienced post-operative complications, which intensified feelings of regret and hindered the process of physical and emotional recovery.

*I believe it was a special case because, as I had complications, I had to be readmitted two days after being discharged, I had to go back to the operating room and have another procedure, and I was in treatment for 60 days. Because of the complications, this period was very difficult.* (P3)

*My surgery had complications. I had to have a video laparotomy two days later, and I was hospitalized in the ICU for approximately 20 days. I believe that because of this, I was not excited about my choice.* (P5)

[...] *I had a post-surgical complication. For 45 days, I really couldn’t eat properly. Food wouldn’t pass from my stomach to my intestines and would always get stuck* [...] *I had to undergo endoscopic dilation, and after that, everything worked fine. At that moment, I felt bad, but after everything was sorted out, the bad feelings went away* [...]. (P10)

On the other hand, some reported excitement and satisfaction with the initial results, particularly in relation to weight loss in the first few weeks:

It was very smooth. I felt good and excited. (P8)

Very good! Despite some limitations imposed by the liquid diet, the weight loss in the first few weeks motivated me a lot. I was sure I had made the right decision. (P12)

The decision to undergo bariatric surgery is not usually made solely in response to medical treatment, but as a move towards rebuilding one’s own being, in the quest to regain autonomy, self-care, and reconnection with the world. Thus, the body ceases to be merely an obstacle and becomes understood as an opening to new ways of being in the world.

### Being in transition: between the strangeness of the body and the psycho-emotional challenges in the post-bariatric period

In the postoperative period, emotional challenges such as compulsive eating and difficulty socializing emerged as expressions of ‘being-in-the-world’ in transition. For many, the transformed body presented itself as a new horizon of possibilities, but also as unknown territory, which demanded constant reconfiguration of being-with and being-there. Binge eating, in this context, can be interpreted as an echo of the internal tensions between the body as an object and the body as an expression of being.

*At Christmas, while my family was having dinner, I felt isolated because I couldn’t eat like them.* (P2)

*When sharing meals with friends, I noticed that many distanced themselves, as if I were sick, which made me feel even more disconnected* [...]*. Many people distance themselves and many invitations are reconsidered because it feels like everyone looks at you with pity, as if you were sick and had been forced to do this, when in fact it was a well-thought-out choice. The feeling is that people don’t understand or know very little about the process.* (P9)

Additionally, the lack of specialized psychological support was cited as a significant difficulty in coping with emotions after surgery.

*My biggest complaint is the lack of psychologists who are knowledgeable about bariatric surgery* [...]*. The ones I tried to see didn’t have any knowledge in this area. I feel a strong urge to eat in the afternoon, but have no help to control this compulsion.* (P5)

Another challenge was the fear of relapse and weight regain, especially during the process of dietary readjustment and lack of physical activity.

*I still have binge eating episodes* [especially when I’m in stressful situations]*, and I always try to take care of myself when I notice it.* (P1)

*I used to eat very quickly, and that got me into trouble three times. I was afraid something had gone wrong.* (P6)

*During the pandemic, when the gyms closed, it was frustrating to see my weight go up*. (P8)

After bariatric surgery, people often experience a range of emotional responses reflecting their existential reconfiguration. From Heidegger’s perspective^(13)^, rapid weight loss and bodily changes are experienced as a transformation of ‘being-in-the-world’. In this sense, the initial anxiety and regret described by the participants can be understood as manifestations of distress in the face of the unknown and new possibilities of existence.

### Reframing the body, rebuilding the being: the transformation of self-esteem after bariatric surgery

Many participants experienced bariatric surgery as a moment of reframing their self-esteem and identity. In Heideggerian terms, these improvements can be understood as a reappropriation of ‘being-in-the-world’, in which the body, previously perceived as an obstacle, emerges as a possibility to assert oneself before the world and others. However, this transformation is complex and not always linear, as aspects such as excess skin and frustrated expectations of the idealized body challenge this new perception of themselves.

*I am very satisfied with my physical body. Being able to buy clothes I never thought I would wear, I feel beautiful and more confident going out in public.* (P5)

*I am a different woman, more empowered, with increased self-esteem, happy, and very satisfied.* (P7)

*After the surgery, my self-esteem improved a lot. I feel more proud, eager to improve my body and image, more confident in relationships, and I see myself better integrated into society [which unfortunately always looks down on obese people]. Better overall well-being.* (P10)

*Even though it has only been two weeks since the surgery, I already see a significant change, which has helped improve my self-esteem*. (P11)

For some, this improvement in self-esteem was accompanied by concerns about excess skin, leading to uncertainty about the need for additional surgery.

*I never had a negative perception of myself* [except in photos]*, but now I love taking pictures and love myself much more. Of course, there are days when I don’t feel very good because of the excess skin* [which bothers me]*, but it’s always temporary.* (P1)

*When I reached my ideal weight, I was shocked by the excess skin and didn’t have the courage to undergo removal surgery. I don’t know if it’s worth the pain and discomfort of the post-surgery period.* (P2)

Many participants stated that the surgery represented a transformation in their lives, providing new opportunities and a more positive self-perception.

*After treating the complication, my life changed a lot. The results began to appear, and I started doing activities that I couldn’t do before or didn’t feel like doing because of my weight.* (P3)

*Literally, I feel like I’m in a new phase. It’s a new life, a new routine. It’s like a rebirth. Although my essence is the same, you have the chance to see yourself with a different perception of the future.* (P9)

*It has improved a lot. The change of clothes, the compliments from people, all of this has a very positive impact. But above all, the increase in physical vigor, mood, energy, and cognitive ability greatly improves my quality of life.* (P12)

*It has improved a lot. For me, the surgery was beneficial. Everything has changed in my life, my energy, memory, sleep, quality of life, everything has improved.* (P13)

These results highlight the complexity of the emotional challenges faced by people undergoing this type of surgery, ranging from frustration and insecurity to a gradual rebuilding of self-esteem and overall well-being. This emotional journey emphasizes the importance of ongoing support, which is essential for facilitating adaptation and promoting a full recovery.

## DISCUSSION

The results of this study highlight the complexity of the challenges people undergoing bariatric surgery face, which go far beyond physical changes. The emotions that emerge during this process range from frustration, insecurity, and fear to hope and, gradually, the rebuilding of self-esteem and well-being. These findings are consistent with the literature, which states that although the physical benefits are undeniable, the emotional, existential, and psychological challenges are equally significant and sometimes underestimated in the clinical follow-up of those who undergo this procedure^(20,21)^.

In Heideggerian phenomenology, understanding this experience requires going beyond a biomedical understanding of obesity and bariatric surgery alone^(13)^. Heidegger invites us to look at human beings not as objects in the world, but as beings-in-the-world, entities in constant relation with the world, with others, and with themselves. Thus, the body is not merely a carrier of biological functions, but the very condition of possibility of existence, of being-in-the-world.

In this context, the limitations imposed by obesity, such as reduced mobility, difficulty finding clothes, stigma, and social exclusion^(22)^, do not merely characterize functional discomforts, but constitute ontological barriers that compromise the realization of the project-of-being. The body comes to be experienced as a body-object, detached from the experience of the lived body, according to the phenomenological distinction. It is this body that, by becoming an obstacle, restricts the possibilities of being and existing in the world.

The decision to undergo bariatric surgery transcends the medical response: it represents an existential movement of self-reappropriation. It involves tensions between the desire for transformation and the fear of the unknown. The anguish experienced is ontological in nature, exposing the self to the loss of references and the opening up of new possibilities of existence^(13)^.

This movement was visible in the participants’ reports, who described feelings of regret, anxiety, and sometimes despair in the immediate postoperative period. This phase represents a suspension of known existential structures, in which the individual needs to rebuild their relationships with themselves, their body, and the world. In this sense, anguish is not only suffering, but a call to authenticity, an impulse to redefine one’s own project of being^(13)^.

The body transformed after surgery ceases to be merely a limitation and becomes a possibility, a fundamental ontological condition for the individual to resume projects that were previously interrupted by obesity. However, this new corporeality is not immediately established, nor is it free of conflict. It requires constant reframing, during which challenges arise, such as feeling strange about one’s own body, discomfort with excess skin, compulsive eating, and tensions in social relationships^(23,24)^.

Here, another central concept in Heidegger’s philosophy becomes evident: care. Being-in-the-world is, in essence, a being of care, going beyond biomedical self-care to encompass an existential dimension, in which the being takes care of itself, others, and the world. It is an ontological movement toward one’s own possibilities^(13)^. In this context, multidisciplinary support is not only technical, but a concrete expression of this care, allowing the person to open up to a new way of being in the world.

The lack of comprehensive care increases the risk of weight regain, behavioral relapses, and psychological distress^(25)^. Thus, post-surgical follow-up should include sensitive listening to existential experiences. Care goes beyond biological normalization and encompasses the lived essence of the person being cared for, with ethical dimensions and recognition of fears and possibilities^(26)^. Participants reported that, despite the challenges, many experienced a sense of existential rebirth, perceiving the body no longer as a burden but as an expression of freedom, which improved their self-esteem, social relationships, and perception of themselves in the world.

In this journey, the role of nursing stands out as an essential dimension of care, not only because it mediates the technical process, but also because it sustains a space for listening, guidance, and welcoming. Nursing, as a practice of care, becomes a co-author in the process of rebuilding patients’ beings, facilitating the transition from a way of being marked by limitation and suffering to another that opens new possibilities^(21,27)^.

Therefore, in light of Heideggerian phenomenology, the experience of bariatric surgery is much more than a physical transformation; it is an ontological phenomenon, in which the being, confronted with its finitude, its losses, and the anguish of the unknown, finds itself called upon to redefine its existence. This process reveals that the body is not a simple object subject to technical intervention, but a fundamental condition of openness to the world, a space for the inscription of projects, relationships, and possibilities of being.

### Study limitations

Remote collection stands out, which may have restricted the diversity of participants.

### Contributions to the fields of Nursing, Health, or Public Policy

The findings reveal that bariatric surgery extends beyond the biomedical realm, representing a profound existential transformation. In this regard, attention is drawn to the need for sensitive nursing care that integrates emotional, social, and existential dimensions, in accordance with the principles of hermeneutic phenomenology. Multidisciplinary follow-up should be continuous and individualized to promote the reconstruction of identity and the reframing of the body. In the field of public health, there is an urgent need for policies that adopt comprehensive, humanized care to address the subjectivity and complexity of obesity and post-surgical phenomena.

## FINAL CONSIDERATIONS

The results of this study reveal that the experience of bariatric surgery extends beyond a physical intervention, constituting an existential transformation. From the perspective of Heidegger’s hermeneutic phenomenology, obesity is not limited to weight gain, but expresses ways of being-in-the-world marked by anguish, limitations, and interrupted projects. In this context, surgery represents not only a medical procedure, but a reconfiguration of the project of being, in which the body, previously experienced as a limitation, becomes a possibility, requiring a reframing of identity, relationships, and existence itself.

This journey reveals the need for a care model that transcends the biological and incorporates emotional, existential, and social dimensions. Multidisciplinary support is essential to help people manage their anxieties, set realistic expectations, and rebuild their relationships with their own bodies and the world.

For future studies, we suggest conducting face-to-face research with larger, more diverse samples, as well as longitudinal approaches that allow us to monitor, over time, the emotional and existential effects of bariatric surgery on people’s lives. From a public policy perspective, it is urgent to incorporate comprehensive care models capable of addressing the complexity of this phenomenon and supporting people in rebuilding their life projects in a full and integrated manner.

## Data Availability

The research data are not available.
