# Supplementary material for: Project-of-being in transformation: existential experience in bariatric surgery in light of phenomenology
Source: Rev Bras Enferm. 2026 Jul 31;79:e20250344. doi: 10.1590/0034-7167-2025-0344 (PMC13435854; doi:10.1590/0034-7167-2025-0344)
Supplement: Translation

# Translation: Projeto-de-ser em transformação: experiência existencial na cirurgia bariátrica à luz da fenomenologia

**DOI:** 10.1590/0034-7167-2025-0344pt

**Authors:** Caroline Linhares de Jesus, Luciara Fabiane Sebold, Bárbara Mohr da Silveira, Lucia Nazareth Amante, Helena Moraes Cortes, Nadia Maria Chiodelli Salum

**Affiliations:** I Universidade Federal de Santa Catarina. Florianópolis, Santa Catarina, Brasil; II Hospital Universtiário Professor Polydoro Ernani de São Thiago. Florianópolis, Santa Catarina, Brasil

**Keywords:** Obesidade, Cirurgia Bariátrica, Enfermagem, Saúde Mental, Fenomenologia.

## Abstract

**Objetivos:**

compreender, à luz da fenomenologia hermenêutica heideggeriana, as experiências existenciais de pessoas submetidas a cirurgias bariátricas.

**Métodos:**

estudo qualitativo, realizado entre abril e julho de 2024, com 13 participantes em diferentes estágios do pós-operatório, recrutados via redes sociais e amostragem pela técnica “bola de neve”. As entrevistas foram conduzidas remotamente e analisadas à luz da fenomenologia hermenêutica de Heidegger.

**Resultados:**

as narrativas indicam que a obesidade é vivida como um limite existencial, que afeta identidade, relações sociais e o ser-no-mundo. A cirurgia surge como possibilidade de ressignificação da existência, mas pode causar compulsões, desafios emocionais e estranhamento com o corpo transformado.

**Conclusões:**

a cirurgia bariátrica é vivida como fenômeno ontológico de transformação do ser. O cuidado multiprofissional, com foco existencial e psicológico, é essencial para apoiar a reconstrução do projeto-de-ser e a relação com o corpo vivido.

## INTRODUÇÃO

Nas últimas décadas, a obesidade consolidou-se como uma questão de saúde pública de caráter pandêmico, que afeta milhões de pessoas em todo o mundo^(1)^. Esse aumento reflete uma interação complexa entre mudanças no ambiente alimentar, sedentarismo, fatores socioeconômicos, ambientais e genéticos, compondo um fenômeno que ultrapassa explicações simplistas^(2)^. Ambientes obesogênicos exercem impacto direto nas escolhas de comportamento e contribuem significativamente para a disseminação dessa condição^(1)^.

Por isso, dada a complexidade, a obesidade demanda uma abordagem integrada e intersetorial, que envolva profissionais da saúde, indústria, organizações sociais e, sobretudo, o poder público, cuja liderança na formulação e implementação de políticas baseadas em evidências é essencial para o enfrentamento eficaz do problema^(3,4)^. Essa articulação é imprescindível para enfrentar os desafios estruturais e promover intervenções sustentáveis, que considerem os determinantes sociais da saúde.

Conforme a Associação Brasileira para o Estudo da Obesidade e Síndrome Metabólica (ABESO) e a Sociedade Brasileira de Endocrinologia e Metabologia (SBEM), a obesidade é caracterizada pelo acúmulo excessivo de gordura corporal, sendo o IMC o principal parâmetro diagnóstico. O sobrepeso corresponde a IMC entre 25,0 e 29,9 kg/m^2^, obesidade a IMC ≥30 kg/m^2^ e obesidade grave a IMC ≥40 kg/m^2^ ou ≥35 kg/m^2^ com comorbidades. Novas diretrizes, porém, propõem complementar essa classificação considerando o maior peso já atingido na vida (MWAL). Essa abordagem valoriza perdas de peso modestas, capazes de proporcionar benefícios clínicos mesmo sem sair da faixa de obesidade, favorecendo um manejo mais individualizado^(5,6)^.

Embora amplamente adotado, o IMC, de forma isolada, não contempla a complexidade do diagnóstico e, portanto, para uma avaliação mais precisa, faz-se necessária a consideração de fatores adicionais, como composição corporal, distribuição de gordura, etnia, presença de comorbidades e histórico clínico, além do uso de métodos complementares, como a bioimpedância^(6-8)^.

O reconhecimento da obesidade como fator de risco para doenças crônicas, como diabetes tipo 2, hipertensão e cardiovasculares, exige, além de mudanças psicossociais, estratégias terapêuticas eficazes^(9,10)^. O tratamento inicia-se com alterações no estilo de vida, incluindo dieta, atividade física, apoio psicológico e, se necessário, uso de medicamentos. Contudo, em casos de obesidade grave sem resposta às abordagens conservadoras, a cirurgia bariátrica é recomendada^(7,11)^.

Tal cirurgia, contudo, não se resume a um procedimento técnico de modificação do trato gastrointestinal. Ela representa uma ruptura ontológica no modo como o sujeito habita o próprio corpo e se relaciona com o mundo^(12)^. As mudanças físicas e metabólicas desencadeiam processos de transformação que atravessam não apenas o corpo biológico, mas também as esferas identitária, afetiva, relacional e existencial. Sob a perspectiva fenomenológica de Heidegger, a obesidade é compreendida como uma experiência que condiciona e molda o ser-no-mundo, com interferências diretas no projeto existencial do sujeito, nas possibilidades de ser e no modo como ele se lança no mundo.

O corpo obeso não é apenas um corpo biológico. Ele é vivido e experimentado como um corpo carregado de significados, muitas vezes associado ao estigma, à inadequação e ao isolamento. As limitações impostas pela mobilidade reduzida, associadas às barreiras sociais e simbólicas, tornam-se vivências que afetam profundamente a constituição do projeto-de-ser dessas pessoas. Heidegger nos adverte que o ser não é dado, mas está sempre em processo, em constante desdobramento, sendo atravessado pela temporalidade, pela historicidade e pelas relações interpessoais^(13)^.

A linearidade causal e a neutralidade científica, nesse contexto, mostram-se insuficientes para explicar a experiência vivida na obesidade e no processo de transformação pós-cirúrgico. A complexidade não reside no objeto em si, mas emerge da relação entre sujeito e mundo, na qual conhecer é, sobretudo, um estar-com, um envolver-se. A cirurgia bariátrica, então, não apenas altera a configuração física do corpo, mas também convoca o sujeito a uma ressignificação da própria existência, atravessando fronteiras entre o corpo vivido e o corpo objetificado.

Por um lado, se a perda de peso favorece a reinserção social, a ampliação da mobilidade e a melhora da saúde, pode, por outro, causar sentimento de estranhamento diante de um corpo que se apresenta como outro, diferente daquele que foi vivido por tanto tempo. Esse processo, frequentemente, traz à tona tensões emocionais, como ansiedade, depressão, insatisfação corporal e, não raro, o surgimento ou agravamento de transtornos alimentares, em decorrência de uma relação fragilizada com a própria imagem e com a alimentação^(14,15)^.

A fenomenologia heideggeriana desloca o olhar da epistemologia para a ontologia, permitindo compreender que a experiência da cirurgia bariátrica não é simplesmente a de um procedimento médico bem-sucedido ou fracassado, mas a de um vir-a-ser em constante construção. Heidegger nos convida a pensar que o ser não é um objeto à disposição da consciência, mas uma possibilidade sempre inacabada, atravessada pelo tempo, pela linguagem e pela abertura ao mundo. Nesse sentido, compreender a experiência da obesidade e da cirurgia é, antes de tudo, abrir-se ao vivido, às formas como os sujeitos se percebem e constroem seus mundos.

Diante desse cenário, o presente estudo, fundamentado na fenomenologia hermenêutica de Heidegger, busca compreender os aspectos emocionais e existenciais vivenciados por pessoas no processo de cirurgia bariátrica, desde a decisão pela intervenção até a adaptação à nova condição de ser-no-mundo. Para tanto, foram elaborados os seguintes questionamentos: Como os pacientes percebem a própria condição de obesidade no contexto de ser-no-mundo? Quais significados emergem durante o processo de decisão e adaptação a esta cirurgia? De que modo as transformações corporais impactam o projeto-de-ser dessas pessoas?

## OBJETIVOS

Compreender, à luz da fenomenologia hermenêutica heideggeriana, as experiências existenciais de pessoas submetidas a cirurgias bariátricas.

## MÉTODOS

### Aspectos éticos

O estudo foi conduzido conforme diretrizes éticas nacionais e internacionais e aprovado pelo Comitê de Ética da Universidade Federal de Santa Catarina, cujo parecer está anexado à presente submissão. Todos os participantes assinaram o Termo de Consentimento Livre e Esclarecido (TCLE) e, para garantir o anonimato e a confidencialidade, foram identificados pela letra “P”, seguida de um número sequencial correspondente à ordem de realização das entrevistas (P1, P2, ..., P13).

### Tipo de Estudo

Estudo qualitativo, fundamentado na fenomenologia hermenêutica heideggeriana (2015). Buscou-se compreender os sentidos e significados atribuídos pelas pessoas à experiência da cirurgia bariátrica, considerando as transformações físicas, emocionais e existenciais que atravessam seus modos de ser-no-mundo. A fenomenologia hermenêutica^(13)^ permite acessar a experiência vivida a partir da compreensão do ser como possibilidade, em constante desdobramento e construção

### Procedimentos metodológicos

Tanto a organização como a descrição do estudo seguiram as diretrizes do *Consolidated Criteria for Reporting Qualitative Research* (COREQ)^(16)^, a fim de garantir transparência, completude e qualidade dos relatos na pesquisa qualitativa, especialmente no que se refere ao desenho metodológico, recrutamento dos participantes, coleta e análise dos dados.

Constituíram critérios de inclusão: pessoas adultas (acima de 18 anos), residentes na capital do estado, que realizaram cirurgia bariátrica em diferentes estágios, mais especificamente durante pós-operatório imediato (até 3 meses), mediato (3 a 12 meses) e tardio (acima de 12 meses), e que estivessem em condições cognitivas e emocionais de participar da entrevista. Foram excluídas pessoas em pré-operatório, bem como aquelas com limitações cognitivas que comprometiam a comunicação verbal e a compreensão das questões.

O recrutamento foi iniciado via Instagram na rede privada do pesquisador, com *post*s e *stories* com convite para participar da pesquisa e *link* para contato via *WhatsApp*, no intuito de assegurar a privacidade. A partir de então, as pessoas que entraram em contato e aceitaram o convite eram solicitadas a indicar outras que também tivessem realizado a cirurgia bariátrica, o que caracteriza a utilização da técnica conhecida como “bola de neve” e favorece a diversidade de experiências. A definição do número de participantes seguiu o princípio da saturação teórica, isto é, a inclusão de novos participantes foi interrompida no momento em que os dados passaram a apresentar recorrência e não emergiam novos elementos relevantes para o aprofundamento da compreensão do fenômeno^(17)^. Compuseram a amostra final 13 participantes, quantitativo considerado suficiente para aprofundamento fenomenológico.

### Cenário do estudo

O estudo foi realizado entre abril e julho de 2024, em uma capital do Sul do Brasil.

### Coleta e organização dos dados

Os dados foram coletados por entrevistas individuais, semiestruturadas, realizadas remotamente via Google Meet, conforme disponibilidade dos participantes. O Termo de Consentimento Livre e Esclarecido (TCLE) foi enviado e explicado previamente pelo *WhatsApp*, assegurando compreensão ética. Utilizou-se um roteiro semiestruturado com 15 questões abertas, com o objetivo de identificar, a partir dos relatos dos participantes, suas experiências pessoais relacionadas à cirurgia bariátrica, inclusive sentimentos positivos e negativos, dificuldades enfrentadas, fatores facilitadores e rede de apoio disponível. As entrevistas, com média de 40 minutos, foram gravadas e transcritas integralmente para análise. A pesquisadora responsável possuía experiência prévia em entrevistas qualitativas, garantindo condução ética e alinhada ao rigor fenomenológico. As entrevistas não foram validadas pelos participantes.

### Análise dos dados

A análise dos dados seguiu os princípios da fenomenologia hermenêutica heideggeriana, a qual busca compreender os modos de ser-no-mundo dos participantes a partir de suas narrativas. Este percurso interpretativo não se limita à descrição dos fenômenos, mas visa o desvelamento dos sentidos que se mostram no discurso, compreendendo o ser como uma possibilidade aberta, em constante relação com o mundo^(13)^.

A análise ocorreu em três etapas: leitura exaustiva das transcrições para apreensão global das experiências; identificação de unidades de significado relacionadas a categorias existenciais como ser-no-mundo, corpo vivido e cuidado; e análise hermenêutica, para articular texto e contexto existencial e, assim, desvelar sentidos profundos conforme a ontologia heideggeriana^(18)^. O olhar fenomenológico hermenêutico pressupõe circularidade na análise, de modo que compreender parte do discurso remete à totalidade da experiência, e esta, por sua vez, ressignifica cada parte, em um movimento conhecido como círculo hermenêutico^(13,19)^.

## RESULTADOS

O estudo contou com a participação de 13 pessoas em diferentes estágios do pós-operatório da cirurgia bariátrica, das quais sete eram mulheres e seis homens, com idades entre 30 e 60 anos. O tempo decorrido desde a realização do procedimento cirúrgico apresentou ampla variação: desde participantes que haviam sido operados há 10 dias até aqueles em processo de recuperação há 8 anos.

Com base na análise dos dados obtidos, emergiram quatro categorias: A compreensão do corpo como limite e possibilidade; Entre a angústia e a esperança: o ser diante das primeiras experiências no pós-operatório bariátrico; O ser-em-transição: entre o estranhamento do corpo e os desafios psicoemocionais no pós-bariátrica; e Ressignificar o corpo, reconstruir o ser: a transformação da autoestima após a cirurgia bariátrica

As categorias que sintetizam os desafios enfrentados nos estágios do perioperatório da cirurgia bariátrica estão ilustradas a seguir por meio dos depoimentos dos participantes. Elas revelam as experiências, com seus significados, em relação à vivência do processo da cirurgia bariátrica.

### A compreensão do corpo como limite e possibilidade

A insatisfação com a imagem corporal foi vivenciada pelos participantes como uma ruptura em suas relações com o mundo, nas quais o corpo obeso se tornou um ‘corpo-objeto’, um obstáculo ao projeto-de-ser. As tentativas frustradas de emagrecimento revelam uma busca por resgatar o controle sobre o próprio corpo e reafirmar a identidade, porém em um processo geralmente permeado por falhas e agudização do sentimento de alienação em relação ao mundo e a si mesmos.

*Frustração por não conseguir perder peso, baixa autoestima, principalmente quando me via em fotos.* (P1)

*Antes da cirurgia, me sentia sempre triste, sem ânimo para fazer nada, várias frustrações por tentar emagrecer e não conseguir, frustrações em datas comemorativas ou festas tipo casamento, onde precisava comprar roupas e nada ficava bom.* (P3)

*Frustração define, não usava as roupas que gostava e sim as que serviam, fora o bullying que sofria, não temos o seu tamanho.* (P7)

Outro aspecto frequentemente mencionado foi a preocupação com a saúde. Alguns atribuíram a decisão de realizar a cirurgia à piora do quadro clínico, o que os conscientizou sobre a urgência da intervenção cirúrgica.

*Inicialmente não queria fazer a tal cirurgia, porém minha saúde física estava piorando e necessitava emagrecer. Coloquei uma meta de um ano, e perda de um quilo por mês, acompanhada de endócrino, nutricionista e psicólogo. Enfim, depois de um ano, não consegui minha meta, então optei pela cirurgia.* (P5)

*Cada vez que eu ia a um médico ou nutricionista e via meu peso aumentando e os exames piorando, eu me via mais sem futuro. Um dos fatores bem relevantes que me fizeram decidir pela cirurgia foi ver que, aos 26 anos, eu tinha as mesmas doenças do meu pai aos 70. Isso foi crucial para minha decisão.* (P9)

*Me sentia com constante falta de energia, problemas de memória, de relacionamento com os meus familiares em razão da minha obesidade e apneia do sono. Com as mudanças que antecederam a minha cirurgia* [abstinência de álcool, rotina de exercícios físicos]*, perdi 8 kg em 1 mês. Isso me deixou ainda mais motivado em realizar a cirurgia.* (P12)

A depressão e o cansaço físico/mental também foram identificados como fatores emocionais presentes antes da cirurgia:

*Sentir-se obeso parecia ser um tabu, não ser muito bem aceito na sociedade ou em relacionamentos. Também me sentia mais depressivo e cansado, mentalmente e fisicamente.* (P10)

É possível compreender, diante das narrativas, que a obesidade não se limita a uma condição meramente biomédica, mas se apresenta como uma experiência existencial marcada por sofrimento, frustração, angústia e, simultaneamente, desejo de transformação. O corpo, vivido como limite, tanto pela insatisfação estética quanto pelas restrições funcionais ou pelos impactos na saúde, também emergiu como possibilidade, na medida em que mobilizou os sujeitos a ressignificarem a própria existência.

### Entre a angústia e a esperança: o ser diante das primeiras experiências no pós-operatório bariátrico

No período imediato após a cirurgia, os participantes experienciaram uma série de emoções conflitantes, que podem ser interpretadas como reflexos da tentativa de abertura do ser-no-mundo. A ansiedade e o medo representam a angústia existencial diante das novas possibilidades e responsabilidades que emergiram com o corpo transformado, enquanto a esperança e as expectativas refletem um movimento em direção a um novo projeto-de-ser.

*Sim, após a cirurgia me arrependi na fase líquida, mas, depois dessa fase líquida, isso passou, fiquei mais nervoso de não poder comer o sólido. Passado um mês, minha mente estava totalmente equilibrada normalmente.* (P2)

[...] *passei por complicações. Então, nos primeiros dois meses, foram apenas sentimentos de arrependimento. Só achava que iria morrer.* (P3)

*Me vi um pouco fragilizada, tive uma sensação de humilhação, tipo eu me vendo precisando de ajuda de outras pessoas e pensava, meu Deus, porque eu não cuidei do meu peso antes. De mim, em geral, para não ter que passar por isso.* (P9)

*As primeiras semanas, digamos uns 30 dias depois, quando comecei a comer sólido, senti que nunca mais iria conseguir comer normalmente. Nesse período você vomita e dói o estômago, daí vem o arrependimento de que nunca mais você vai conseguir comer sólidos sem passar perrengue, mas é passageiro.* (P10)

Alguns participantes enfrentaram complicações pós-operatórias, o que intensificou sentimentos de arrependimento e dificultou o processo de recuperação física e emocional.

*Acredito que seja um caso à parte, pois, como eu tive complicações, precisei internar novamente após dois dias de alta, precisei voltar ao centro cirúrgico e reabordar, fiquei em tratamento por 60 dias. Por conta da complicação, esse período foi bem difícil.* (P3)

*Minha cirurgia teve complicações. Tive que fazer uma laparotomia de vídeo com dois dias, e fiquei internada na UTI por aproximadamente 20 dias. Acredito que, por conta disso, não estava animada com minha escolha.* (P5)

[...] *tive uma intercorrência pós-cirúrgica, com 45 dias realmente não estava conseguindo comer direito, não passava a comida do estômago para intestino, entalava sempre* [...] *tive que fazer uma dilatação via endoscópica e, após isso, tudo funcionou bem. Nesse momento me senti ruim, mas depois que tudo se ajeitou passou os sentimentos ruins* [...]. (P10)

Por outro lado, alguns relataram empolgação e satisfação com os primeiros resultados, particularmente em relação à perda de peso nas primeiras semanas:

*Foi bem tranquilo, me senti bem, empolgado.* (P8)

*Muito bem! Apesar de algumas limitações impostas pela dieta líquida, a perda de peso nas primeiras semanas me motivou muito. Estava seguro de que havia tomado a decisão correta.* (P12)

A decisão pela cirurgia bariátrica, geralmente, não se dá apenas como resposta a um tratamento médico, mas como um movimento de reconstrução do próprio ser, na busca por recuperar a autonomia, o cuidado de si e a reconexão com o mundo. Assim, o corpo deixa de ser apenas um obstáculo e passa a ser compreendido como abertura para novas formas de ser-no-mundo.

### O ser-em-transição: entre o estranhamento do corpo e os desafios psicoemocionais no pós-bariátrica

No período pós-operatório, os desafios emocionais, como a copulsão alimentar e a dificuldade de socialização emergiram como expressões de ‘ser-no-mundo’ em transição. Para muitos, o corpo transformado apresentou-se como um novo horizonte de possibilidades, mas também como um território desconhecido, que demandou reconfiguração constante do ser-com e o ser-aí. A compulsão alimentar, nesse contexto, pode ser interpretada como um eco das tensões internas entre o corpo enquanto objeto e o corpo enquanto expressão do ser.

*No Natal, enquanto minha família jantava, eu me senti isolado, pois não podia comer como eles.* (P2)

*Ao compartilhar refeições com amigos, percebi que muitos se afastaram, como se eu estivesse doente, o que me fez sentir ainda mais desconectado* [...]. *Muitas pessoas se afastam e muitos convites são repensados porque a sensação que dá é que todo mundo te olha com pena, como se você tivesse doente e tivesse sido obrigada a fazer isso, sendo que foi uma escolha bem pensada. O sentimento é de que as pessoas não entendem ou sabem muito pouco sobre o processo.* (P9)

Adicionalmente, a falta de apoio psicológico especializado foi citada como uma dificuldade significativa no enfrentamento das emoções após a cirurgia.

*Minha maior queixa é a falta de psicólogos competentes sobre a bariátrica* [...]. *Os que tentei ir não têm conhecimento na área. Sinto bastante vontade de comer à tarde, mas sem ajuda para controlar essa compulsão.* (P5)

Outro desafio foi o medo de recaídas e reganho de peso, especialmente durante o processo de readaptação alimentar e ausência de atividades físicas.

*Ainda tenho crises de compulsão alimentar* [principalmente quando estou em situações de estresse] *e tento sempre me cuidar quando percebo!* (P1)

*Tinha o hábito de comer muito rápido e isso me deixou em situações complicadas três vezes, tive medo de ter dado algo errado.* (P6)

*Na pandemia, quando fecharam as academias, foi uma frustração ver o peso subir.* (P8)

Após a cirurgia bariátrica, as pessoas costumam experimentar uma gama de respostas emocionais que refletem a própria reconfiguração existencial. Sob a ótica de Heidegger^(13)^, a rápida perda de peso e as mudanças corporais são vividas como uma transformação do ‘ser-no-mundo’. Nesse sentido, a ansiedade inicial e o arrependimento descritos pelos participantes podem ser compreendidos como manifestações de angústia diante do desconhecido e das novas possibilidades de existência.

### Ressignificar o corpo, reconstruir o ser: a transformação da autoestima após a cirurgia bariátrica

A cirurgia bariátrica foi vivenciada por muitos participantes como um momento de ressignificação da autoestima e da própria identidade. No referencial heideggeriano, essas melhorias podem ser entendidas como uma reapropriação do ‘ser-no-mundo’, na qual o corpo, antes percebido como um entrave, emerge como uma possibilidade para afirmar-se diante do mundo e dos outros. No entanto, essa transformação é complexa e nem sempre linear, pois aspectos como o excesso de pele e as expectativas frustradas com o corpo idealizado desafiam essa nova percepção de si mesmos.

*Estou muito satisfeita com meu corpo físico, poder comprar roupas que nunca pensei em vestir, me sinto bonita e com mais confiança para frequentar lugares públicos.* (P5)

*Sou outra mulher, mais empoderada, autoestima aumentada, feliz e muito satisfeita.* (P7)

*Após a cirurgia minha autoestima melhorou muito, me sinto mais vaidoso, com vontade de melhorar o corpo e a imagem, mais confiança para relacionamentos e melhor me vejo inserido na sociedade* [que infelizmente sempre tem um olhar mais “de lado” para pessoas obesas]*. Bem-estar geral melhor.* (P10)

*Mesmo com apenas duas semanas de cirurgia, já vejo uma mudança significativa e isso já tem ajudado a melhorar a autoestima.* (P11)

Para alguns, essa melhora na autoestima foi acompanhada de preocupações com o excesso de pele, o que gerou insegurança sobre a necessidade de novas cirurgias.

*Nunca tive uma percepção ruim de mim mesmo* [menos em fotos]*, mas agora adoro tirar fotos, estou me amando muito mais. Claro que tem dias que não me sinto muito bem por conta do excesso de pele* [que incomoda]*, mas sempre é passageiro.* (P1)

*Quando atingi o peso ideal, me assustei com o excesso de pele e não tenho coragem de me submeter à cirurgia de remoção. Não sei se vale a pena as dores e desconfortos do pós-cirúrgico.* (P2)

Muitos participantes afirmaram que a cirurgia representou uma transformação em suas vidas, por proporcionar novas oportunidades e uma percepção mais positiva de si mesmos.

*Após o tratamento da complicação minha vida mudou muito, os resultados começaram a aparecer, comecei a realizar atividades que antes não conseguia ou não sentia vontade por conta do peso.* (P3)

*Literalmente, me sinto em uma nova fase, é uma nova vida, nova rotina, é como um renascimento. Embora minha essência seja a mesma, você tem a chance de se enxergar com outra percepção de futuro.* (P9)

*Melhorou muito. A mudança de roupas, os elogios das pessoas, tudo isso tem um impacto muito positivo. Mas, principalmente, o aumento do vigor físico, humor, disposição e capacidade cognitiva melhoram muito a minha qualidade de vida.* (P12)

*Melhorou muito. Para mim, a cirurgia foi benéfica. Tudo mudou em minha vida, disposição, memória, sono, qualidade de vida, tudo melhorou.* (P13)

Esses resultados ressaltam a complexidade dos desafios emocionais enfrentados por pessoas submetidas a esse tipo de cirurgia, que vão desde frustrações e inseguranças até uma gradual reconstrução da autoestima e do bem-estar geral. Essa trajetória emocional enfatiza a importância de um suporte contínuo, essencial para facilitar a adaptação e promover uma recuperação integral.

## DISCUSSÃO

Os resultados deste estudo evidenciam a complexidade dos desafios vivenciados por pessoas submetidas a cirurgias bariátricas, que vão muito além das transformações físicas. As emoções emergentes nesse processo oscilam entre frustração, insegurança, medo, esperança e, gradualmente, reconstrução da autoestima e do bem-estar. Esses achados estão em consonância com a literatura, segundo a qual, embora os benefícios físicos sejam inegáveis, os desafios emocionais, existenciais e psicológicos são igualmente significativos e, por vezes, subestimados no acompanhamento clínico de quem se submete a este procedimento^(20,21)^.

Na fenomenologia heideggeriana, compreender essa experiência exige ultrapassar o entendimento biomédico da obesidade e da cirurgia bariátrica unicamente^(13)^. Heidegger nos convida a olhar para o ser humano não como um objeto no mundo, mas como um ser-no-mundo, um ente em constante relação com o mundo, com os outros e consigo mesmo. Assim, o corpo não é um mero portador de funções biológicas, mas a própria condição de possibilidade do existir, de ser-no-mundo.

Nesse horizonte, as limitações impostas pela obesidade, como mobilidade reduzida, dificuldade de encontrar roupas, estigma e exclusão social^(22)^, não caracterizam apenas desconfortos funcionais, mas se configuram como barreiras ontológicas, que comprometem a realização do projeto-de-ser. O corpo passa a ser vivido como um corpo-objeto, desvinculado da experiência do corpo vivido, segundo a distinção fenomenológica. É esse corpo que, ao se tornar obstáculo, restringe as possibilidades de ser e estar no mundo.

A decisão pela cirurgia bariátrica transcende a resposta médica: representa um movimento existencial de reapropriação do ser. Envolve tensões entre o desejo de transformação e o medo do desconhecido. A angústia vivida possui caráter ontológico, ao expor o ser à perda de referências e à abertura de novas possibilidades de existência^(13)^.

Esse movimento foi visível nos relatos dos participantes, que descreveram sentimentos de arrependimento, ansiedade e, por vezes, desespero no pós-operatório imediato. Essa fase representa uma suspensão das estruturas existenciais conhecidas, nas quais o ser precisa reconstruir suas relações consigo mesmo, com o corpo e com o mundo. A angústia, nesse sentido, não é apenas sofrimento, mas um chamado à autenticidade, um impulso para a ressignificação do próprio projeto-de-ser^(13)^.

O corpo transformado após a cirurgia deixa de ser apenas limite e passa a ser também possibilidade, condição ontológica fundamental para que o ser retome projetos outrora interrompidos pela obesidade. Contudo, essa nova corporeidade não se constitui de forma imediata, tampouco isenta de conflitos. Exige um trabalho constante de ressignificação, no qual surgem desafios como o estranhamento diante do próprio corpo, o desconforto com o excesso de pele, a compulsão alimentar e as tensões nas relações sociais^(23,24)^.

Aqui, evidencia-se outro conceito central em Heidegger: o cuidado. O ser-no-mundo é, por essência, um ser de cuidado, indo além do autocuidado biomédico para abarcar uma dimensão existencial, na qual o ser se ocupa de si, dos outros e do mundo. É um movimento ontológico em direção às próprias possibilidades^(13)^. Nesse contexto, o suporte multiprofissional não é apenas técnico, mas expressão concreta desse cuidado, permitindo à pessoa abrir-se a um novo modo de ser-no-mundo.

A falta de cuidado integral eleva o risco de reganho de peso, recaídas comportamentais e sofrimento psíquico^(25)^. Assim, o acompanhamento pós-cirúrgico deve incluir escuta sensível às experiências existenciais. O cuidado ultrapassa a normalização biológica e abrange a essência vivida do ser cuidado, com dimensões éticas e reconhecimento de medos e possibilidades^(26)^. Os participantes relataram que, apesar dos desafios, muitos vivenciaram sensação de renascimento existencial, percebendo o corpo não mais como peso, mas como expressão de liberdade, o que melhorou a autoestima, as relações sociais e a percepção de si no mundo.

Neste percurso, destaca-se o papel da enfermagem como uma dimensão essencial do cuidado, não apenas por mediar o processo técnico, mas por sustentar um espaço de escuta, orientação e acolhimento. A enfermagem, como prática do cuidado, torna-se coautora no processo de reconstrução do ser dos pacientes, ao facilitar a transição de um modo de ser marcado pela limitação e pelo sofrimento para outro que se abre a novas possibilidades^(21,27)^.

Portanto, à luz da fenomenologia heideggeriana, a experiência da cirurgia bariátrica é muito mais do que uma transformação física; é um fenômeno ontológico, no qual o ser, confrontado com sua finitude, com suas perdas e com a angústia do desconhecido, se vê convocado a ressignificar sua existência. Esse processo revela que o corpo não é um simples objeto passível de intervenção técnica, mas condição fundamental de abertura ao mundo, espaço de inscrição dos projetos, das relações e das possibilidades de ser.

### Limitações do estudo

Destaca-se a coleta remota, que pode ter restringido a diversidade dos participantes.

### Contribuições para a área da Enfermagem, Saúde ou Política Pública

Os achados revelam que a cirurgia bariátrica ultrapassa o aspecto biomédico, pois representa uma profunda transformação existencial. Nesse sentido, alerta-se para a necessidade de um cuidado de enfermagem sensível, capaz de integrar dimensões emocionais, sociais e existenciais, consoante com os princípios da fenomenologia hermenêutica. O acompanhamento multiprofissional deve ser contínuo e individualizado, no sentido de promover a reconstrução da identidade e ressignificação do corpo. No âmbito da Saúde Pública, reforça-se a urgência de políticas que adotem cuidado integral e humanizado, para contemplar a subjetividade e a complexidade do fenômeno da obesidade e do pós-cirúrgico.

## CONSIDERAÇÕES FINAIS

Os resultados deste estudo desvelam que a experiência da cirurgia bariátrica vai além de uma intervenção física, configurando-se como um processo de transformação existencial. Sob a ótica da fenomenologia hermenêutica de Heidegger, a obesidade não se limita ao acúmulo de peso, mas expressa modos de ser-no-mundo atravessados por angústias, limitações e projetos interrompidos. A cirurgia, nesse contexto, não representa apenas um procedimento médico, mas uma reconfiguração do projeto-de-ser, na qual o corpo, antes vivenciado como limite, passa a ser possibilidade, exigindo a ressignificação da identidade, das relações e da própria existência.

Esse percurso revela a necessidade de um modelo de cuidado que transcenda o biológico, a fim de incorporar dimensões emocionais, existenciais e sociais. O acompanhamento multiprofissional é fundamental para auxiliar as pessoas na elaboração das angústias, na gestão das expectativas e na reconstrução da relação com o próprio corpo e com o mundo.

Para estudos futuros, sugere-se o desenvolvimento de pesquisas presenciais, com amostras mais amplas e diversificadas, além de abordagens longitudinais que permitam acompanhar, ao longo do tempo, os efeitos emocionais e existenciais da cirurgia bariátrica na vida das pessoas. Do ponto de vista das políticas públicas, torna-se urgente a incorporação de modelos de cuidado integral, capazes de acolher a complexidade desse fenômeno e apoiar as pessoas na reconstrução de seus projetos-de-ser de forma plena e integrada.

## Data Availability

Os dados de pesquisa não estão disponíveis.
